# Carrier Induced Hopping to Band Conduction in Pentacene

**DOI:** 10.1038/s41598-019-56558-w

**Published:** 2019-12-27

**Authors:** Varsha Rani, Pramod Kumar, Akanksha Sharma, Sarita Yadav, Budhi Singh, Nirat Ray, Subhasis Ghosh

**Affiliations:** 1 School of Physical Sciences, Jawaharlal Nehru University, New Delhi, 110067 India; 2 Inter-University Accelerator Center, Aruna Asaf Ali Marg, New Delhi, 110067 India

**Keywords:** Applied physics, Materials for devices

## Abstract

Charge transport in organic thin films which are generally polycrystalline is typically limited by the localization of the carriers at lattice defects resulting in low carrier mobilities and carriers move from one state to another state by hopping. However, charge transport in organic semiconductors in their single crystalline phase is coherent due to band conduction and mobilities are not limited by disorder resulting in higher carrier mobility. So it is a challenge to enhance the carrier mobility in a thin film which is the preferred choice for all organic devices. Here, we show that it is possible to increase the carrier mobility in polycrystalline thin films by injecting sufficient carriers such that Fermi level can be moved into the region of high density in Gaussian density of states of molecular solids. When the hopping transport happens through the molecular energy levels whose density is low, mobility is decided by incoherent transport however, when the the hopping transport happens through the energy levels with high density, mobility is decided by coherent transport, as in band conduction. We present results highlighting the observation of both band-like and hopping conduction in polycrystalline organic thin films by varying the concentration of injected charge. More importantly the transition from hopping to band transport is reversible. The observed carrier mobilities in both the regimes match well with theoretical estimates of hopping mobility and band mobility determined from first principles density functional theory.

## Introduction

Low values of charge carrier mobilities in organic thin films is a fundamental issue affecting their applications to integrated circuits, displays and memory devices requiring fast processing^1^. Unlike single crystals where band-like transport is observed^2–5^, intrinsic thermal and structural disorder in organic thin films lead to localization of charge carriers in the tail of a Gaussian density of states (GDOS)^6,7^ which results due to randomness in positional and energetic disorder in polycrystalline and amorphous thin films. Carriers then need some activation energy to hop between neighboring sites leading to (i) low carrier mobility and (ii) positive temperature coefficient of mobility. The real challenge with polycrystalline organic thin films lies in achieving delocalized band-like conduction resulting in high mobility comparable with that in pure organic single crystals. Although, delocalized charge transport with high mobilities has been observed in pure single crystal^3,5^ however, organic single crystals cannot be used in large area rolable and foldable electronic devices. Poor device integration and cross-talk between devices are also the drawbacks of single crystals. Considerable efforts have been put^8,9^ towards improving the performance of organic thin films based devices by engineering the growth parameters, substrate/organic interface and device parameters but, high mobilities comparable with that in single crystals could not be achieved. Observation of negative temperature coefficient in polycrystalline thin films have previously been observed and interpreted in terms of large thermal fluctuations in electronic coupling rather than band transport^10–12^. Hence, an understanding of the crossover from localized to delocalized band-like transport in polycrystalline organic thin film is essential. We show that temperature coefficient of mobility can be changed from positive to negative corresponding to a transition from hoping to band-like transport as the Fermi level is modulated by injecting sufficient numbers of carriers. The observed values of carrier mobilities in the band transport regime are two orders of magnitude higher than those in the hoping regime. Our experimental results are supported by theoretical calculations for hoping mobility estimates using Marcus theory^13,14^ and density functional theory (DFT) band mobility.

## Results and Discussion

**Figure 1:**
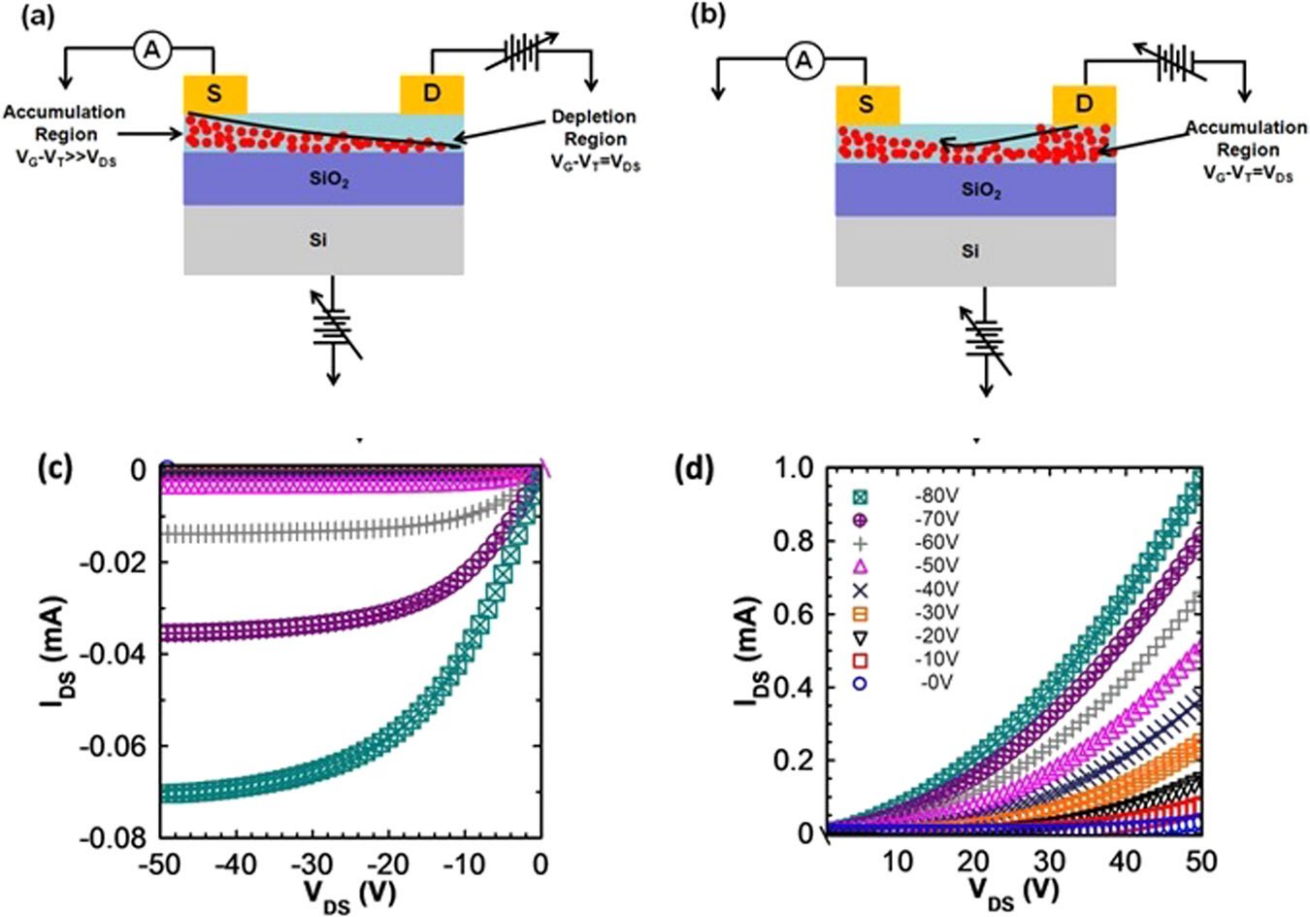
Schematic representation of the working and output characterstics of a p-type OFET: (**a**) Negative *V*_*DS*_ regime and (**b**) positive *V*_*DS*_ regime. In negative *V*_*DS*_ regime, two regions exist within the channel; accumulation region and depletion region. However, in positive *V*_*DS*_ regime, there is no depletion region rather only accumulation region exists for the whole channel. (**c**,**d**) Show the room temperature output characteristics (*I*_*DS*_ − *V*_*DS*_) of pentacene thin film based OFET in negative and positive *V*_*DS*_ regime, respectively. Mechanisms of charge transport in negative and positive *V*_*DS*_ regime seem different.

Figure 1 illustrates the working of a pentacene thin film based organic field effect transistor (OFET) in negative as well as positive source-drain bias (*V*_*DS*_) regime. A negative gate voltage (*V*_*G*_) accumulates a layer of holes at organic/dielectric interface. When a negative *V*_*DS*_ is applied, charge carriers flow from source to drain and two different regions; accumulation region near source and depletion region due to the pinch off the channel near drain exist (schematically show in Fig. 1(a)). However, in positive *V*_*DS*_ regime, working of the OFET is different from that in negative *V*_*DS*_ regime. When a positive bias is applied at drain, device structure becomes quite similar to a hole only two terminal device (Fig. 1(b)). There are however two key differences; (i) organic thin film is sandwiched laterally between two metal electrodes whereas it is sandwiched vertically in two terminal devices, and (ii) an additional *V*_*G*_ is used to control the density of background charge carriers in the device. In positive *V*_*DS*_ regime, as drain is at higher potential than source, holes are injected at the drain electrode and move towards source i.e. source and drain are interchanged. Further, in this regime, as there is no pinch-off, no depletion region is observed within the channel rather only accumulation region exists for the whole channel. Figure 1(c,d) represent the resulting output (*I*_*DS*_ − *V*_*DS*_) characteristics of pentacene thin film based OFET fabricated under optimized growth conditions (*see* Fig. S1 in Supplementary Information for morphological and structural data), in negative and positive *V*_*DS*_ regime, respectively. In negative *V*_*DS*_ regime, *I*_*DS*_ − *V*_*DS*_ exhibits two regions; linear region at small *V*_*DS*_ and saturation region at high *V*_*DS*_ due to the pinch-off the channel. However, in positive *V*_*DS*_ region, as there is no pinch-off, no saturation is observed in *I*_*DS*_ − *V*_*DS*_ characteristics rather *I*_*DS*_ increases non-linearly with *V*_*DS*_ (Fig. 1(d)). Further, magnitudes of *I*_*DS*_ in positive *V*_*DS*_ regime are higher by more than one order in comparison to those in negative *V*_*DS*_ regime. *I*_*DS*_ increases with *V*_*G*_ in both, negative as well as positive *V*_*DS*_ regime due to an increase in density of free holes. The crux of the matter is how to operate OFET so that Fermi level can be sufficiently moved up in the high density of states region from the region with low density of states in GDOS.

**Figure 2:**
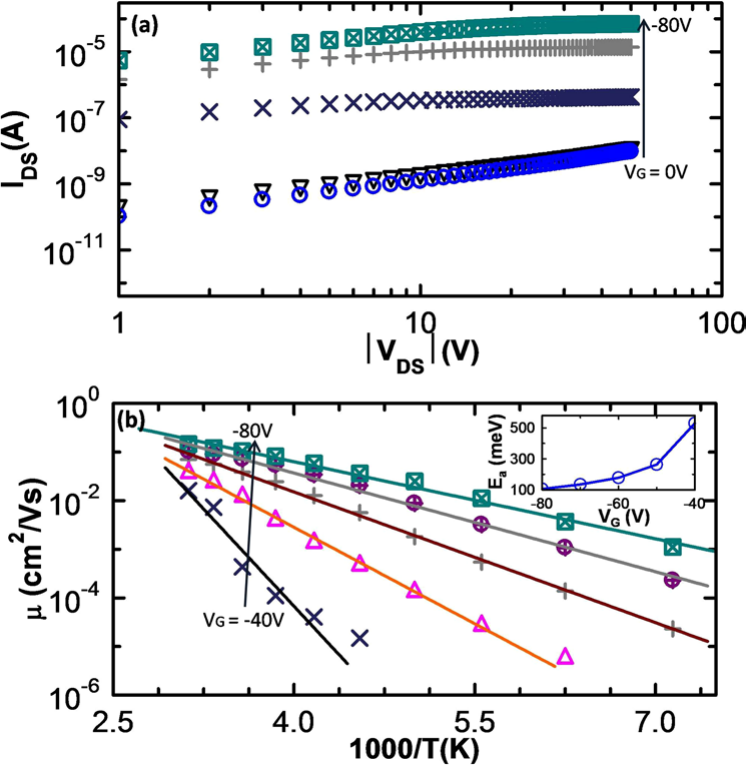
Negative source−drain regime: (**a**) *I*_*DS*_ − *V*_*DS*_ on a log-log plot for a pentacene thin film based OFET. Here, linear and saturation regions are clearly distinguishable. (**b**) Arrhenius temperature dependence of charge carrier mobility measured at different *V*_*G*_. Inset shows the variation of activation energy with the *V*_*G*_. Different symbols represent different values of the applied *V*_*G*_.

**Figure 3:**
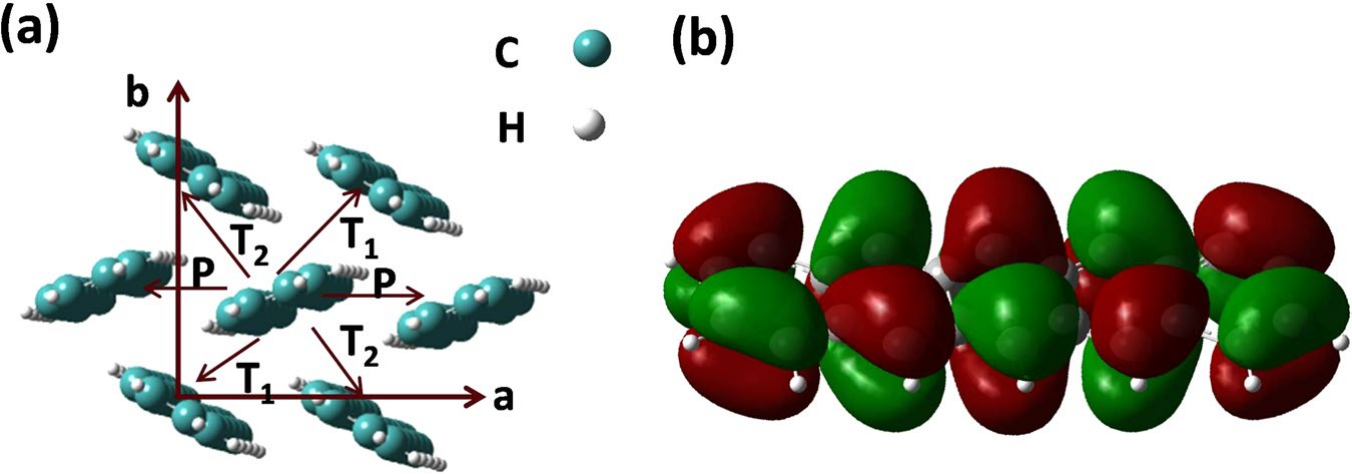
Dimer representation: (**a**) Schematic representation of different dimers in ab-plane; *P* dimers along a-axis, *T*_1_ and *T*_2_, transverse dimers along diagonals and (**b**) isosurface representing the charge density distribution on HOMO of pentacene.

Figure 2(a) shows the *I*_*DS*_ as a function of the negative |*V*_*DS*_| on a log-log plot. Two regions; linear and saturation are observed clearly in *I*_*DS*_ − *V*_*DS*_. We estimate the field-effect mobility, *μ*, by fitting the *I*_*DS*_ − *V*_*DS*_ characteristics in the linear region as^15^: ${I}_{DS}=\mu \frac{W}{L}{C}_{i}[({V}_{G}-{V}_{T}){V}_{DS}-\frac{{V}_{DS}^{2}}{2}]$, where *C*_*i*_ is the dielectric capacitance per unit area and *V*_*T*_ is the threshold voltage. The room temperature mobility has been found to be 0.3 cm^2^/Vs which is at least two order of magnitude lower than that in single crystalline pentacene, matching well with the typical mobilities observed in other studies^16–19^. Figure 2(b) shows the variation of the extracted carrier mobilities with temperature and *V*_*G*_. We find that at all *V*_*G*_, mobilities increase with temperature exhibiting Arrhenius behavior ($\mu \, \sim \,exp(\,-\,{E}_{a}/{k}_{B}T)$, *E*_*a*_ being the activation energy, *k*_*B*_, Boltzmann’s constant and T, the temperature). This is consistent with thermally activated hopping transport. Inset of Fig. 2(b) shows that at higher *V*_*G*_, charge carriers require lesser activation energy to hop to neighboring sites. This is attributed to the filling of the higher energy states by additional charge carriers at high *V*_*G*_. In other words, Fermi level shifts from the tail towards the center of the GDOS. Next, we compare the experimentally extracted hopping mobility with that estimated theoretically using Marcus theory^13^. Within this approach, the charge transfer between neighboring molecular sites is described as a self-exchange reaction process. First diffusion coefficient, (*D*) is calculated using the charge transfer rate (*k*_*ij*_) as^20^

1 $$D=\frac{1}{2n}\sum _{j}\,{r}_{j}^{2}{k}_{ij}{P}_{j};\,{k}_{ij}=\frac{4{\pi }^{2}}{h}{t}_{ij}^{2}\frac{\exp (-\,\lambda \mathrm{/4}{k}_{B}T)}{\sqrt{4\pi \lambda {k}_{B}T}}$$

and then hopping mobility is estimated using the Einstein relation as,

2 $$\mu =\frac{eD}{{k}_{B}T}$$

where, *n* is the space dimensionality, *r*_*j*_, hopping distance, *e*, the electronic charge, *h*, the Planck’s constant, ${P}_{j}(\,=\,{k}_{ij}/\sum {k}_{ij})$ is hopping probability of a charge carrier corresponding to the hopping pathway, *j*, the transfer integral, *t*_*ij*_ represents the strength of the coupling between neighboring sites, *i* and *j* and *λ*, the intramolecular reorganization energy arises due to the relaxation of the geometry of a molecule during charge transfer. This is estimated by carrying out single point energy calculations on optimized geometries of neutral and ionic molecule as^21^ $\lambda =[({E}_{\pm }^{\text{'}}-{E}_{\pm })+({E}^{\text{'}}-E)]$. Here *E* and *E*_±_ are the ground state energies of the molecule in the neutral state and in the ionic state (‘+’ and ‘−’ signs represent the cation and anion, respectively) respectively. *E*^′^ is the energy of the neutral molecule in the optimized geometry of the charged molecule and ${E}_{\pm }^{\text{'}}$ is the energy of the charged molecule in the optimized geometry of the neutral molecule. *t*_*ij*_ have been estimated by employing dimer projection method^22^, directly from the orbitals of dimers and monomers. Values of *λ*, *t*_*ij*_ and *μ* calculated in this way are found to be; *λ* = 97 meV, *t*_*ij*_ for different dimers lying in ab-plane Fig. 3(a)), *i.e*., *P*, *T*_1_ and *T*_2_ = 57.74, 65.38 and 68.46 meV, and *μ* = 1.8 cm^2^/Vs. Figure 3(b) shows the distribution of charge density on the highest occupied molecular orbital (HOMO) of pentacene. We observe that HOMO is uniformly distributed over the all the benzene rings in pentacene. Further, as H-atoms have no p-orbitals, they do not contribute to the *π*-orbitals of HOMO. Predicted magnitudes of *λ*, *t*_*ij*_ and *μ* are comparable with that estimated in previous studies on pentacene^23–25^. The theoretically calculated mobility estimates are higher than the experimentally observed values. This can be attributed to the thermal and energetic disorder in neighboring molecules that have not been included in theoretical model.

It can be emphasized here that this should be the highest mobility that can be achieved in hopping regime in a-b plane whereas along c-axis maximum mobility has been found to be 4.45 × 10^−5^ *cm*^2^/*Vs*. Actually, c-axis should be the relevant direction for charge transport in two-terminal sandwiched devices. For comparison, we also studied Al/pentacene/Au based two terminal devices (For details, see Figs. S2 and S3 in Supplementary Information). Mobilities in these devices have been estimated using space charge limited conduction (SCLC) method^6^ and found to be 1.21 × 10^−5^ *cm*^2^/*Vs*, close to the theoretically predicted value. This can be attributed to the minimal effect of disorder on transport along c-axis compared to that in a-b plane as evident from the excellent matching of experimental and theoretical values of mobility along c-axis compared to difference (0.3 vs 1.8 cm^2^/Vs) in a-b plane.

**Figure 4:**
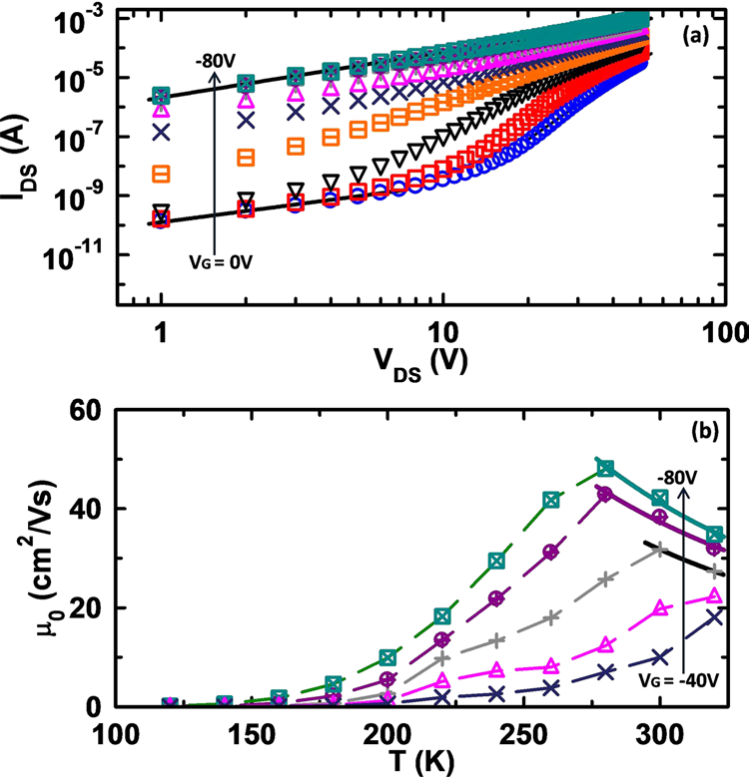
Positive drain – source regime: (**a**) *I*_*DS*_ − *V*_*DS*_ on a log-log plot for a pentacene thin film based OFET. *V*_*DS*_ − *I*_*DS*_ characteristics are similar to two terminal devices *i.e*. initial Ohmic region and then field dependent SCLC. However as *V*_*G*_ increases SCLC starts weakening and approaches Ohmic region for the entire range of *V*_*DS*_. (**b**) Temperature dependence of charge carrier mobility measured at different *V*_*G*_ in positive *V*_*DS*_ regime. Dashed lines are used to connect the different data points at same *V*_*G*_ and solid lines represent fitting according to the power-law ($\mu (T)\, \sim \,{T}^{-\gamma }$) at high temperatures. Different colored symbols represent different values of the *V*_*G*_.

Figure 4(a) shows the *I*_*DS*_ as a function of positive *V*_*DS*_, on a log-log plot. We observe that magnitudes of current in this regime are higher by more than one order of magnitude than those in negative *V*_*DS*_ regime. At low *V*_*DS*_, the carriers injected from one electrode redistribute themselves in such a way that they replace the holes flowing out at the other end. Hence charge transport is contact limited in which *I*_*DS*_ is governed by the thermally generated free charge carriers in the device and varies linearly with *V*_*DS*_ resulting in an ohmic region. At high bias, the injected carrier density becomes large resulting in accumulation of holes, which create a space charge region. The current then shows non-linear behavior with *V*_*DS*_ consistent with space charge limited conduction (SCLC)^6^. As expected, no saturation in current is observed because channel is not pinched-off in this regime and hence, no depletion region forms.

With increasing *V*_*G*_, the background charge carrier concentration in the device increases. At higher *V*_*G*_s, more carriers are injected from the source. This weakens the SCLC due to enhanced conduction through the channel. Thus SCLC weakens and eventually a crossover to ohmic conduction is observed, as shown in Fig. 4(a). As the output characteristics of the device in this regime are different from those in negative *V*_*DS*_ regime hence, conventional transistor method cannot be applied to extract mobility. *I*_*DS*_ − *V*_*DS*_ characteristics shown in Fig. 4(a) have been simulated by solving the set of equations, *I*_*DS*_ = *ep*(*x*)*μ*[*T*, *F*]*F*(*x*)*A*, $dF(x)/dx=ep(x)/{\varepsilon }_{s}$ and ${V}_{DS}={\int }_{0}^{L}\,F(x)dx$, self consistently. The same simulation has been used to extract the mobility in two-terminal devices and as mentioned before, excellent corroboration of experimental data with first principle theoretical data is observed. Here *A* is the effective area of cross-section, ${\varepsilon }_{s}$ is the dielectric constant of organic semiconductor and *p(x)* is the density of charge carriers at a distance *x* from the injecting electrode. Room temperature mobility estimated in this way has been found to be 48.3 cm^2^/Vs at a *V*_*G*_ of −80 *V* which is almost two orders of magnitude higher than that in the negative *V*_*DS*_ regime and also much higher than the hopping mobility limit set by Marcus theory. And the most importantly this value is close to mobiilities generally obtained in pentacene single crystal^3^.

Figure 4(b) shows the variation of *μ* with temperature and *V*_*G*_, estimated in positive *V*_*DS*_ regime. We observe that mobilities increase with temperature up to ~280 K, consistent with charge transport limited by impurity scattering^15,26^. Beyond ~280 K, the decrease in mobility with an increase in temperature can be explained on the basis of phonon scattering^15,26^. After fitting the mobility curves in the high temperature range according to $\mu (T)\, \sim \,{T}^{-\gamma }$, *γ* has been found to be in the range of 2.1–2.4. These values of *γ* match well with those obtained for pure organic crystals^2,27^, further emphasizing the role of phonon scattering in polycrystalline organic thin films. The role of thermal fluctuations in intermolecular electronic coupling as suggested by *Troisi et al*.^10^ is ruled out by carrying out molecular dynamics simulations. We observe no signature of band-like transport from these simulations.

**Figure 5:**
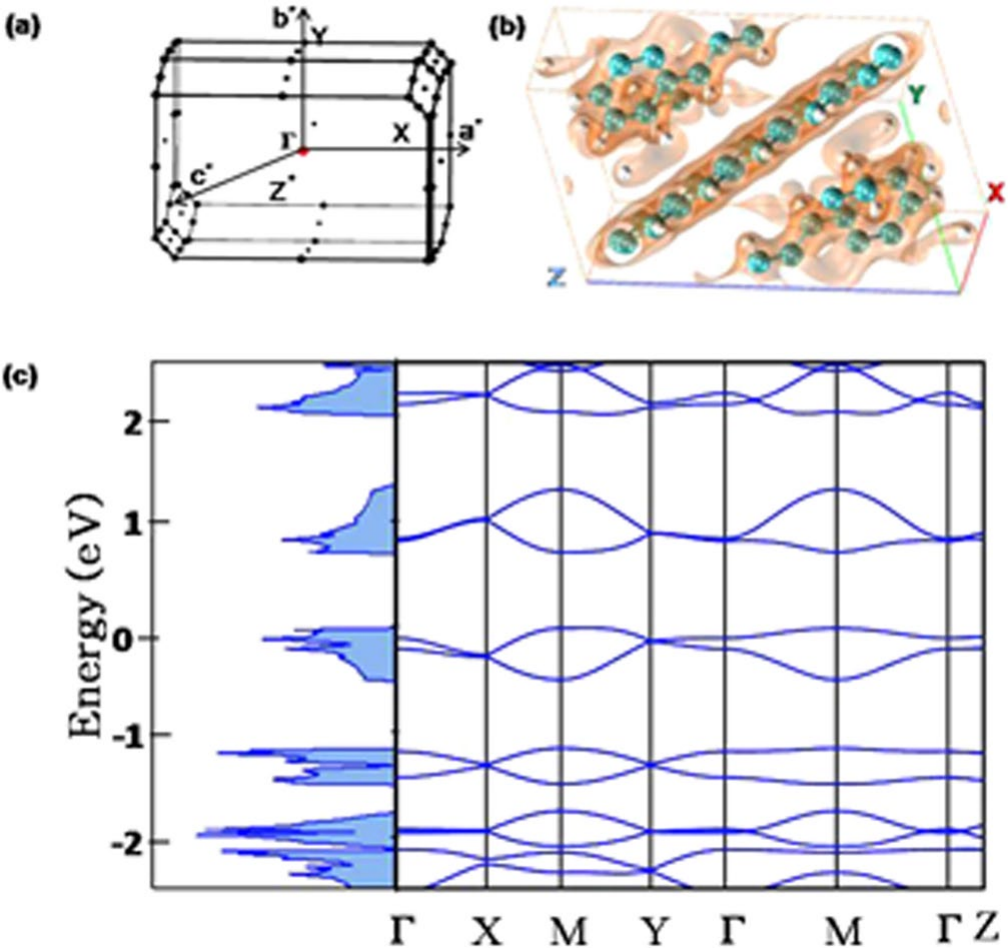
Density functional theory calculations: (**a**) First Brillouin zone for crystal structure of pentacene in reciprocal lattice. (**b**) Distribution of charge density in the surrounding of molecules in pentacene unit cell. (**c**) Calculated band structure and density of states of pentacene using periodic DFT calculations. Different high symmetry points are represented in units of (2*π*/*a*, 2*π*/*b*, 2*π*/*c*) along certain directions as Γ = (0, 0, 0), X = (0.5, 0, 0), M = (0.5, 0.5, 0), Y = (0, 0.5, 0) and Z = (0, 0, 0.5). Zero of energy has been set at the valence band maximum.

Further, as mentioned before, the room temperature mobility estimated from *I*_*DS*_ − *V*_*DS*_ in positive *V*_*DS*_ regime is consistent with the band mobility values expected for single crystals^3^. To support our experimental observations of band transport, we have theoretically estimated the band mobilities of pentacene bulk phase^28,29^ using periodic DFT based calculations. Brillouin zone of pentacene with high symmetric *k*-points used in the calculation of band structure are shown in Fig. 5(a). Figure 5(b) shows the distribution of charge density in the surrounding of molecules in pentacene unit cell. Figure 5(c) shows the band structure and density of states for pentacene bulk phase^29^. The band structure calculations have been carried out in Brillouin zone connecting the different high symmetry points, X, M, Y, Z with the internal coordinates being (0, 0, 0), (0.5, 0, 0), (0.5, 0.5, 0), (0, 0.5, 0) and (0, 0, 0.5) in units of (2*π*/*a*; 2*π*/*b*; 2*π*/*c*), respectively. As there are two molecules in equivalent configuration in pentacee unit cell, each band in band structure is composed of two subbands. Pentacene shows a direct band gap at Γ to be 0.70 eV which is lower than the experimentally observed one (2.2 eV)^30^. This is a common drawback of DFT however, it does not affect the accuracy of transport parameters estimated from band structure because, transport parameters are estimated from the slope of the valence or conduction band and not from the band gap.

From band structure, carrier mobility can be estimated as^24^,

3 $$\mu =\frac{e\tau }{{m}_{e}}$$

so there are two main parameters to be determined; effective mass (*m*_*e*_) and relaxation time (*τ*). Effective mass has been calculated from band structure as^24^, ${m}_{e}={\hslash }^{2}{(\frac{{d}^{2}E(k)}{d{k}^{2}})}^{-1}$. The relaxation time has been estimated using the acoustic deformation potential and follows as^31^,

4 $$\tau =\frac{{\hslash }^{2}B}{{(2\pi {k}_{B}T)}^{\mathrm{1/2}}{m}^{\mathrm{1/2}}{D}_{p}}$$

where *B* is the bulk modulus and *D*_*p*_ is the accoustic deformation potential. *B* and *D*_*p*_ can be given by^31^,

5 $$B={V}_{0}{(\frac{{\partial }^{2}E}{\partial {V}^{2}})}_{{V}_{0}}$$

*V*_0_ being the equilibrium volume of the unit cell and

6 $${D}_{p}=\frac{1}{{V}_{0}}\frac{\partial {E}_{VC}}{\partial (\Delta V)}$$

where Δ*V* represents the change in the volume of pentacene unit cell and *E*_*vc*_ is the energy difference between the core level and top of maximum valence band for hole transport. As localized 1 s level is not sensitive to the slight lattice deformation, it can be used as energy reference to obtain the absolute band energy change for valence band maximum (VBM). This energy difference between the VBM and 1 s level is plotted against the fractional change in volume to obtain *D*_*p*_. Then band mobility, estimated using Eq. 3 has been found to be 55.52 *cm*^2^/*Vs* which is higher than our experimental value (48.34 cm^2^/Vs). The difference in experimental and theoretical values can be accounted if structural disorder is included in the calculation, which is extremely difficult and beyond the scope of present problem.

Our results then suggest that the crossover from hopping to band-like transport occurs due to the large variation in the injected carrier concentration in negative and positive *V*_*DS*_ regime. To confirm this, we have analytically estimated the carrier concentration and corresponding Fermi level in both regimes. The total charge concentration in the channel at a spatial position *x* (distance from the injecting electrode) can be written as:

7 $${p}_{tot}(x)={p}_{f}+{p}_{G}+{p}_{DS}(x)$$

where *p*_*f*_ is the concentration of thermally generated free charge carriers and *p*_*G*_ and *p*_*DS*_(*x*) are the charge carrier concentrations injected by the gate and *V*_*DS*_, respectively. In the negative *V*_*DS*_ regime, when *V*_*G*_ is larger than the *V*_*DS*_, *p*_*tot*_, using gradual channel approximation can be written as^32^,

8 $${p}_{tot}(x)={p}_{f}+{C}_{i}({V}_{G}-\frac{x}{L}{V}_{DS})/et$$

where *t* is the thickness of the accumulation layer and has been take to be 10 nm. When *V*_*DS*_ approaches the *V*_*G*_, channel is depleted and the Fermi level lies in the deep localized states below the equilibrium level (−*σ*^2^/*k*_*B*_*T*, *σ* being the width of GDOS), resulting into low values of mobility^6^.

In the positive *V*_*DS*_ regime, at low bias, injected carrier concentration is equal to the extracted one and the transport is injection limited. Then total carrier concentration and position of the Fermi level is decided by the *V*_*G*_ i.e.

9 $${p}_{tot}={p}_{0}+{C}_{i}{V}_{G}/et$$

At high bias, all the charge carriers injecting at one electrode are not balanced by those extracting at the other, resulting accumulation of charge carriers (SCLC). Then *p*_*DS*_(*x*) can be expressed as (see Supplementary Information S4)^15,33^,

10 $${p}_{DS}(x)=3{V}_{DS}{\varepsilon }_{s}{x}^{-1/2}/4{L}^{3/2}$$

and the total carrier concentration is given by Eq. 7. Dependence of the Fermi level on the carrier concentration is given by^7^,

11 $${\int }_{-\infty }^{\infty }\,f(E,{E}_{F})g(E)dE={p}_{tot}$$

where *f*(*E*, *E*_*F*_) = [1 + exp(*E*_*F*_ − *E*)/*k*_*B*_*T*]^−1^, is Fermi function, exhibiting the energetic distribution of the charge carriers at thermal equilibrium. *g*(*E*) represents the GDOS in HOMO of the organic semiconductor given by^7^,

12 $$g(E)=({N}_{V}/\sqrt{2\pi }\sigma )\,{\exp }\,(-\frac{{E}^{2}}{2{\sigma }^{2}})$$

*N*_*V*_ is effective density of states in the HOMO. Hence, position of *E*_*F*_ depends on three parameters; (i) spatial position (x) between source and drain (ii) magnitude of negative or positive *V*_*DS*_ i.e. |*V*_*DS*_| and (iii) magnitude of *V*_*G*_. Lets examine the effect of each of these parameters separately.

**Figure 6:**
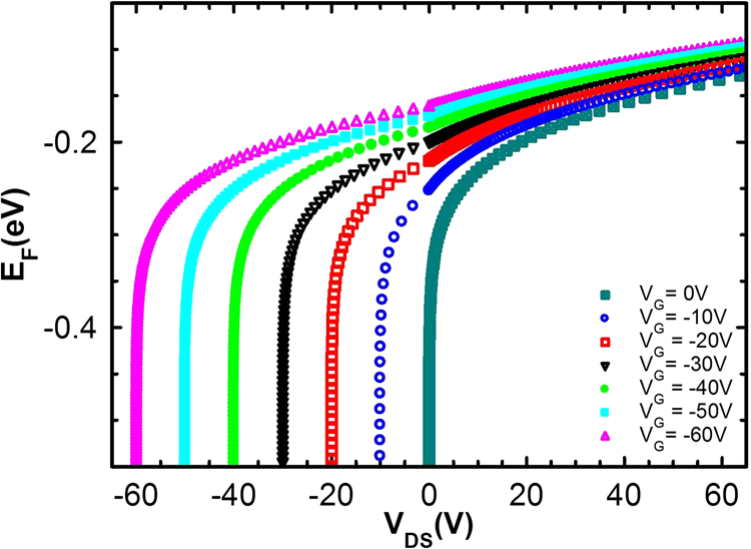
Estimated positions of Fermi-energy level: Estimated Fermi level (*E*_*F*_) as a function of *V*_*DS*_ at different |*V*_*G*_|.

**Figure 7:**
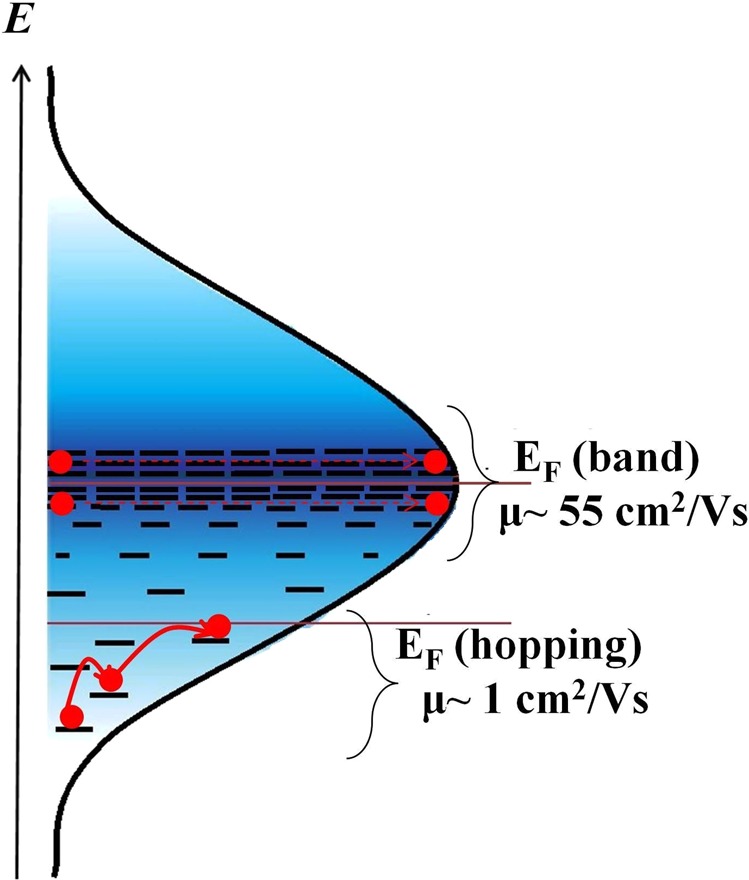
Schematic representation of charge transport in GDOS: Schematic illustration of charge transport governed by two different sections of the GDOS. When the Fermi level lies near the tail of the GDOS, transport is described by thermally activated hopping. As the Fermi level moves towards the central region, band-like transport becomes possible.

Estimated positions of Fermi level in negative and positive *V*_*DS*_ regime have been represented in Fig. 6. In the negative *V*_*DS*_ regime, the Fermi level goes down and becomes flat after the channel is pinched-off. Hence there are two regions in negative *V*_*DS*_ regime: accumulation and depletion. The density of occupied states is high in the accumulation region and low in the depletion region, resulting in the current being controlled by the low mobility region i.e. the tail of the GDOS. In the positive *V*_*DS*_ regime, as there is no depletion region, the Fermi level across the entire channel remains near the center of the GDOS and the current is governed by the high mobility region (see Figs. S4 and S5 in Supplementary Information). This would explain the band-like transport with higher mobilities in positive *V*_*DS*_ regime in pentacene thin film based OFETs. Figure 7 schematically illustrates the charge transport, governed by two different sections of the GDOS. When the Fermi level lies near the tail of the GDOS, transport is described by thermally activated hopping. As the Fermi level moves towards the central region, band-like transport becomes possible. Table 1 summarizes the mobilities obtained in two-terminal and three-terminal devices. we observe that mobilities in two-terminal devices are lower by several orders of magnitude than those obtained in three-terminal devices. This large difference in mobilities in different device configuration is attributed to the anisotropy in the coupling between the dimers along different axis.

**Table 1:** Summary of experimentally observed mobility (*μ*_*exp*_) and theoretically estimated mobility (*μ*_*th*_) in two-terminal (2T) and three-terminal (3T) devices.

		*μ*_*exp*_ (*cm*^2^/*Vs*)	*μ*_*th*_ (*cm*^2^/*Vs*)	Mechanism of charge transport
2T		1.21 × 10^−5^	4.45 × 10^−5^	Hopping
3T	−ve *VDS*	0.32	1.84	Hopping
(OFET)	+ve *V*_*DS*_	48.34	55.52	Band

In conclusion, we have shown that both, hopping and band-like transport can be achieved in same organic system by varying the concentration of injecting carriers. This has been achieved by exploiting the presence and absence of pinch-off region by changing the polarity of source and drain in FETs. Band-like conduction associated with high mobility has been observed in positive *V*_*DS*_ regime due to the high concentration of injected carriers which moves the Fermi level near the center of the GDOS. Here, density of localized states is so high that charge carriers require negligible activation energy to move to neighboring sites and act like a wave, resulting in band-like transport. It is then also conceivable that this approach can possibly be used to access high mobility regimes in the broader class of disordered semiconductors and insulators.

## Methods

### Experimental details

We choose pentacene for our study, due to its relatively high carrier mobility in polycrystalline thin film and single crystal form^3,14^. High purity (>99.999%), triple sublimed pentacene has been thermally evaporated to grow thin films. For two terminal devices, 200 nm single layer of pentacene is sandwiched between Al and Au. We fabricate three terminal OFETs with Au source, drain contacts on a 100 nm thick pentacene film grown on n^++^ Si/SiO_2_ substrate. All the thin films have been grown at a base pressure of 5 × 10^−6^ mbar with an extremely low evaporation rate of 0.1 Å/s to minimize structural disorder^9^. The channel length, *L* and width, *W* are fixed at 20 *μ*m and 3 mm, respectively.

### Computational details

Theoretical estimates for the hopping mobility have been calculated using Gaussian 09^34^, by employing ground state energy calculations on pentacene monomer and dimers using a B3LYP (Becke, three-parameter, Lee-Yang-Parr) functional^35,36^ and 631-G (d, p) basis set^37^. Transfer integrals are calculated using versatile Object-oriented Toolkit for Coarse-graining Applications (VOTCA) package^38,39^. We take into account the non-orthonormality of frontier orbitals of monomers and estimate the transfer integrals by projecting monomer orbitals on the dimer orbitals^22^. We estimate the band mobility using periodic DFT calculations using Quantum Espresso package^40^, within the generalized gradient approximation with Perdew-Burke-Ernzerhol (PBE)^41^ exchange correlation function and ultrasoft pseudopotentials. Dispersion interactions between organic molecules are taken into account by using London type pairwise empirical atomic interactions as implemented by DFT-D2 method^42,43^.

## Supplementary information

Supplementry information.
