# Supplementary material for: Carrier Induced Hopping to Band Conduction in Pentacene
Source: Sci Rep. 2019 Dec 27;9:20193. doi: 10.1038/s41598-019-56558-w (PMC6934786; doi:10.1038/s41598-019-56558-w)
Supplement: Supplementary file 1 — Supplementry information. [file 41598_2019_56558_MOESM1_ESM.pdf]

Supplementary Information

Carrier Induced Hopping to Band Conduction in Pentacene

Varsha Rani,[1] *Pramod Kumar,*[1] *Akanksha Sharma,*[1] *Sarita Yadav,*[1] *Budhi Singh,*[2] *Nirat Ray,*[1] *Subhasis Ghosh*[1]*

[1]School of Physical Sciences, Jawaharlal Nehru University, New Delhi 110067, India
[2]Inter-University Accelerator Center, Aruna Asaf Ali Marg, New Delhi 110067, India
E-mail: subhasis.ghosh.jnu@gmail.com

S1. Morphological and structural characterization of pentacene thin films

To examine the effect of growth parameters on surface morphology and crystalline quality, we have fabricated thin films of pentacene at different substrate temperatures (T_G) and with a rate of 0.1 Å/s. At low evaporation rate, incoming molecules get enough time to find out a preferred orientation resulting ordered growth with minimum structural disorders. Top row of Fig. S1 shows the surface morphologies of pentacene thin films grown at different T_G. At room temperature (30°C), surface morphology of pentacene exhibits layer by layer and upward pyramidal growth. This type of growth is modeled by combining horizontal diffusion limited aggregation (DLA) type with vertical mound growth.[1] As T_G increases, island size starts reducing however, their layered structure remains intact upto T_G = 70°C (middle row shows height profiles along the red lines drawn in top row). At 100°C, ordered growth greatly disturbs and thin film consists of small and randomly oriented crystallites.

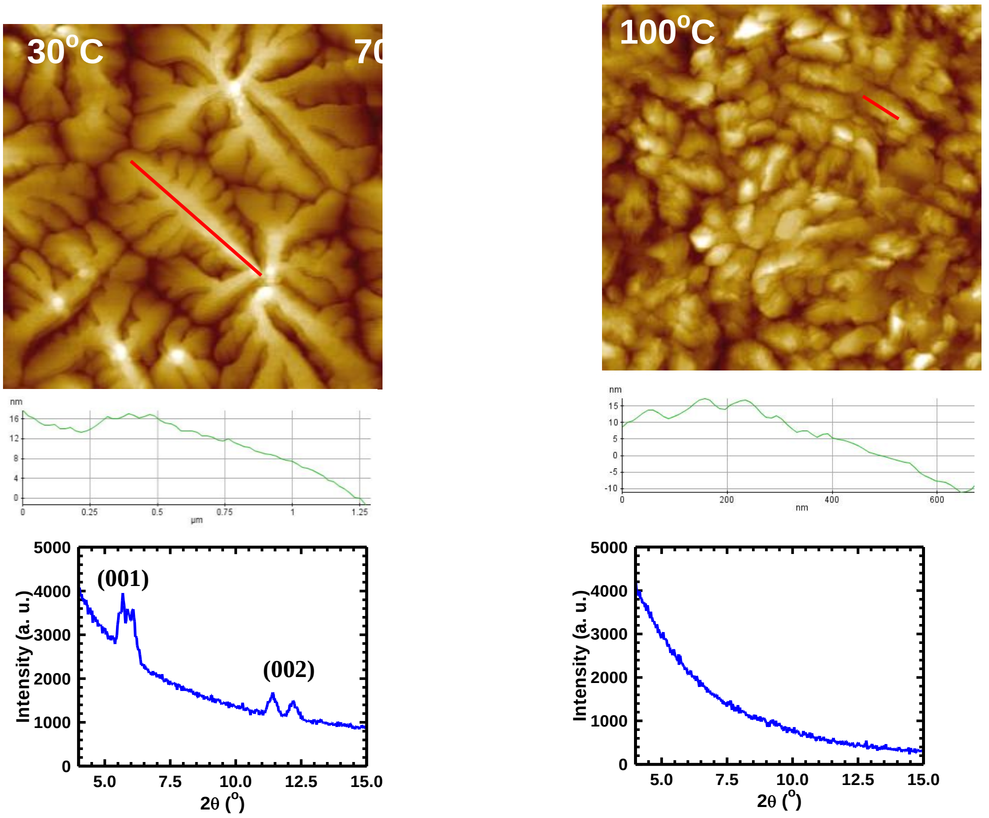

Figure S1. AFM topographic images (scan size 5μm X 5μm) of 100 nm pentacene thin films deposited on SiO_2 substrates at different substrate temperatures and at deposition rate 0.1 Å/s (Top row). Middle and bottom rows show respective height profiles along the red line drawn in top row and X-ray diffraction patterns for corresponding thin films, respectively.

Thus nucleation rate increases with temperature and islands transform from pyramidal at low T_G to small crystallites with large number of grain boundaries at high T_G. These grain

boundaries act as traps for the charge carriers and hinder the charge transport between source and drain in pentacene thin film based organic field effect transistors (OFETs).

Bottom row shows the corresponding X-ray diffraction (XRD) patterns of pentacene thin films. XRD pattern of thin film grown at room temperature exhibits two diffraction peaks at $2\theta = 5.7^o$ and 11.4^o corresponding to the plane (001) and (002).[2] It means that a-b plane lies parallel to the substrate. Further, average interplanar spacing has been estimated to be 1.54 nm, consistent with the c-axis of unit cell of pentacene in thin film phase[2] i.e. c-axis lies almost perpendicular to the substrate. Other low intensity peaks at $2\theta = 6.09^o$ and 12.2^o result the interplanar spacing to be ~ 1.45 nm, consistent with the bulk phase of pentacene.[3] Hence, pentacene thin film exhibits the contributions due to both phase i.e. thin film and bulk phase. However, thin film phase dominates the bulk phase. As T_G increases, crystallinity of the film starts reducing. At 70^oC, intensity of first order peak reduces and high order peaks disappear. As T_G increases further, all the peaks get disappeared i.e. crystallites are randomly oriented.

S2. Electrical characterization

(a) Current-voltage (*J-V*) and capacitance-voltage (*C-V*) characteristics of Al/pentacene/Au based Schottky diodes

Pentacene is a hole transport material with highest occupied molecular orbital (HOMO) at 5.0 eV and lowest unoccupied molecular orbital (LUMO) at 2.9 eV. The work functions of Au and Al are 5.2 eV and 4.2 eV, respectively.[4] There would be no barrier at Au/pentacene interface but, a barrier of 0.8 eV exists for hole at Al/pentacene interface. Figure S2(a) shows the current-voltage (*J-V*) characteristics of Al/pentacene/Au based Schottky diode. Inset shows the charge transport between two pentacene molecules in two-terminal devices. The current due to the hole injection from positively biased Au was measured and then the current due to hole injection from Al was measured by reversing the polarity of the bias voltage *V*. *J-V* characteristics of Al/pentacene/Au device display asymmetric behavior. The current injected from Au electrode is higher by five orders of magnitude than that from Al electrode. Further, at low positive bias (V ≤ 1), current shows exponential dependence on applied bias. At higher bias, dependence of current on voltage changes from exponential to power-law type and current in two terminal device is controlled by space charge limited conduction (SCLC).[4,5] In this region, *J-V* characteristics are fitted with Mott Gurney square law

$$J = \frac{9}{8}\mu(T)\varepsilon_s\frac{(V-V_{bi})^2}{d^3} \qquad (1)$$

where $\mu(T)$ is the mobility of charge carriers and depends strongly on temperature due to hopping transport, d is the thickness of the pentacene thin film, ϵ_s is the dielectric constant of organic semiconductor and V_{bi} is the built-in potential due to the difference in the work functions of two metal electrodes. $\mu(T)$, estimated by fitting *J-V* characteristics according to Eq. 1, has been found to be 1.21 x 10^{-5} cm^2/Vs.

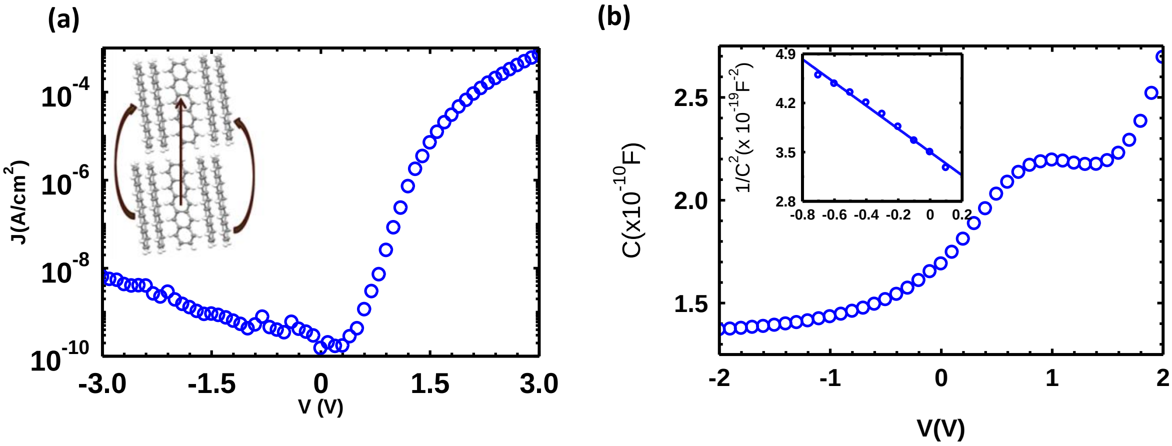

Figure S2. (a) *J-V* characteristics of Al/pentacene/Au based Schottky diode. Inset represents the direction of charge transport in two terminal devices. (b) *C-V* characteristics of Al/pentacene/Au based Schottky diodes. Insets show $1/C^2$-V characteristics in reverse bias and the linear dependence confirms the formation of depletion region in reverse bias.

Figure S2(b) shows the capacitance-voltage (*C-V*) characteristics of the same Al/pentacene/Au Schottky diode. In the reverse bias (V < 0), capacitance decreases as the magnitude of the applied bias (|V|) increases whereas in forward bias region (0 < V < 1),

capacitance increases with voltage. This decrease and increase in capacitance with reverse and forward bias, respectively, confirms the presence of depletion region due to band bending.[6] In this region, capacitance C_I of a Schottky diode as a function of V can be written as[7],

$$C_I = \left(\frac{0.5\, A^2 e \in_s N_A}{V_{bi} \pm V}\right)^{1/2} \qquad (2)$$

where A is the area of the device, e is the electronic charge and N_A is the acceptor concentration. Positive and negative signs give the capacitance in reverse and forward bias, respectively. According to Eq. 2, if we plot $1/C^2$ as a function of applied bias in region I then it should be a straight line. $1/C^2$ -V plot has been shown in the inset of Fig. S2(b) and a straight line also confirms the band bending and presence of depletion region in Al/pentacene/Au diode. When V becomes equal to V_{bi} depletion region is collapsed and the situation is known as the flat band condition. Under this condition depletion capacitance diminishes. Then a peak in *C-V* characteristics should be observed at V_{bi}, due to the combined effect of dropping of the depletion capacitance and subsequently increase of the diffusion capacitance.[8] In Fig. S2(b), a peak due to the collapse of depletion region has been observed at $V = 1 \pm 0.05$ V in *C-V* characteristics. Hence, from *C-V* characteristics V_{bi} is obtained to be 1.0 ± 0.05V which is equal to the difference in work function of two metals, Au and Al. This further illustrates that intrinsic properties of organic molecules (chemical structure etc.) do not play any role in determining the V_{bi}. Except, electronic energy levels (HOMO and LUMO) of organic molecules and metal work functions need to be engineered, in order to fabricate a perfect Schottky diode. Also, growth conditions of thin film need to be optimized, to avoid the extra localization of charge carriers by structural disorder induced defects at metal/molecule interface.

S3. Arrangement of pentacene molecules in thin film

As is clear from the XRD pattern of pentacene thin film shown in the bottom row of Fig. S1 that a-b plane lies parallel to the substrate, hence, charge transport in OFETs should be governed by this plane. Whereas c-axis that lies almost perpendicular to the substrate should be main direction of charge transport in two-terminal sandwiched devices. Figure S3(a) and S3(b) show the arrangement of pentacene molecules in two terminal sandwiched devices and in three terminal devices (OFET), respectively. Characterization of OFET has been presented in main article. Maximum mobility (μ_{max}), estimated in each device geometry has also been summarized in figure.

Transport along c-axis

(Two terminal devices)

Transport in a-b plane

(Three terminal devices)

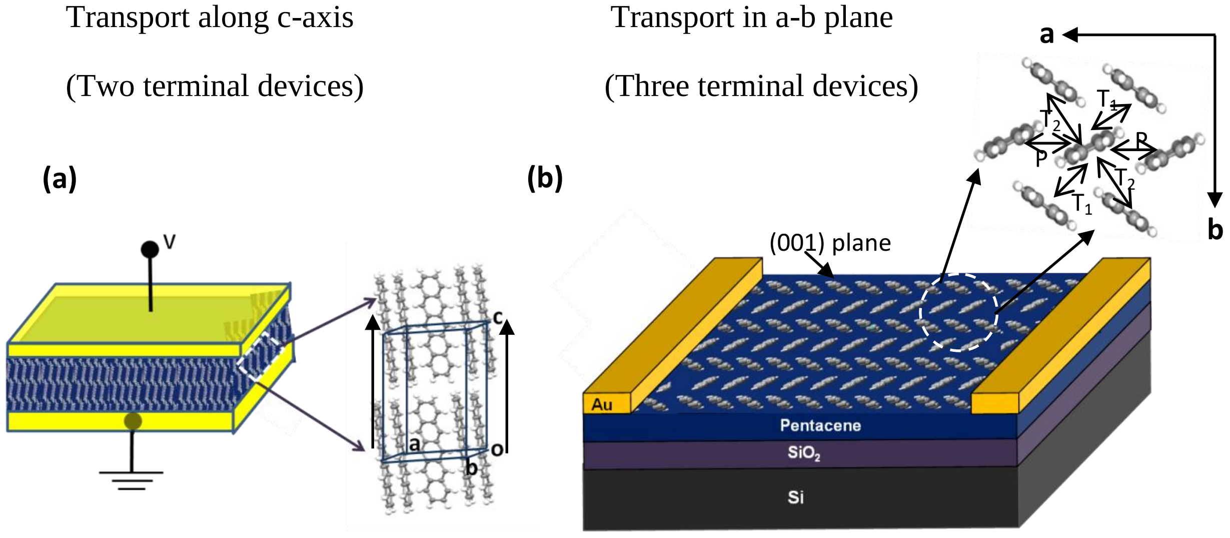

μ_{max} = 4.45 x 10^{-5} cm^2/Vs

μ_{max} (Hopping conduction) = 1.84 cm^2/Vs

μ_{max} (Band conduction) = 55.52 cm^2/Vs

Figure S3. Schematic representation of arrangement of pentacene molecules (a) along the direction perpendicular to the substrate and (b) in a plane parallel to the substrate. These are relevant planes for charge transport in two and three terminal devices, respectively.

We observe that μ_{max} in three terminal devices is higher by several orders of magnitude than that in two terminal sandwiched devices. Hence charge transport in pentacene thin film is highly anisotropic. This anisotropy in μ_{max} must be attributed to the intrinsic anisotropy in the efficiency of coupling i.e. transfer integral (t_{ij}) between neighboring molecules existing along different directions. As charge transport in two terminal devices can be treated as one dimensional i.e. along c-axis and t_{ij} between the molecules along c-axis is quite low (2.6 meV). Hence, mobility in two terminal devices is also low. Whereas in three terminal devices, charge transport takes place in a plane (a-b plane) and pentacene molecules adopt herringbone arrangement with a stacking along a-axis. Further, there are three dimers in a-b plane; parallel dimers (P) along a-axis and transverse dimers (T_1 and T_2) along diagonals. Average t_{ij} in a-b plane has been estimated to be 63.8 meV, leading to high mobility in OFETs.

S4. Carrier concentrations injected by positive V_{DS}

The total charge concentration in the channel at a spatial position x (distance of the injecting electrode) can be written as

$$p_{tot}(x) = p_f + p_G + p_{DS}(x)$$

Here, p_f is the concentration of thermally generated free charge carriers and are the charge carrier concentrations injected by the V_G and V_{DS}. In the negative V_{DS} regime, when the V_G is larger than the source-drain bias, p_{tot}, using gradual channel approximation can be written as,

$$p_{tot}(x) = p_f + \frac{C_i\left(V_G - \frac{x}{L}V_{DS}\right)}{et}$$

t is the thickness of the accumulation layer and has been taken to be 10 nm.

In the positive V_{DS} regime, at low bias, injected carrier concentration is equal to the extracted one and the transport is injected limited. Then total carrier concentration and the position of the Fermi level is decided by the gate voltage i.e.

$$p_{tot}(x) = p_o + \frac{C_i V_G}{et}$$

At high bias, all the charge carriers injecting at one electrode are not balanced by those extracting at the other, resulting accumulation of charge carriers (SCLC). Then $p_{DS}(x)$ may be estimated as follows. In SCLC regime, μ is estimated using Eq.

$$I_{DS} = ep(x')\mu[T, F(x')]A$$

and

$$\frac{dF(x')}{dx'} = \frac{ep(x')}{\epsilon_S}$$

$$I_{DS} = \mu[T, F(x')]\epsilon_S A F(x')\frac{dF(x')}{dx'}$$

after integrating spatially from 0 to x and putting boundary condition ($F(0) = 0$), we get,

$$F(x) = \sqrt{\frac{2I_{DS}x}{\mu[T, F(x')]\epsilon_S}}$$

In steady state, I_{DS} should be independent of x and can be given by the equation,

$$I_{DS} = \frac{9}{8}\mu\epsilon_S\frac{V_{DS}^2}{L^3}$$

Putting this value of I_{DS} in above $F(x)$ and solving for $F(x)$ we get,

$$F(x) = \frac{3}{2} x^{\frac{1}{2}} \frac{V_{DS}}{L^{\frac{3}{2}}}$$

Finally, putting the value of $F(x)$ in Eq, $p(x)$ can be given as

$$p(x) = \frac{3V_{DS}\epsilon_S x^{-\frac{1}{2}}}{4L^{\frac{3}{2}}}$$

This actually the P_{DS} and is written as

$$P_{DS}(x) = \frac{3V_{DS}\epsilon_S x^{-\frac{1}{2}}}{4L^{\frac{3}{2}}}$$

S5. Working of a p-type OFET

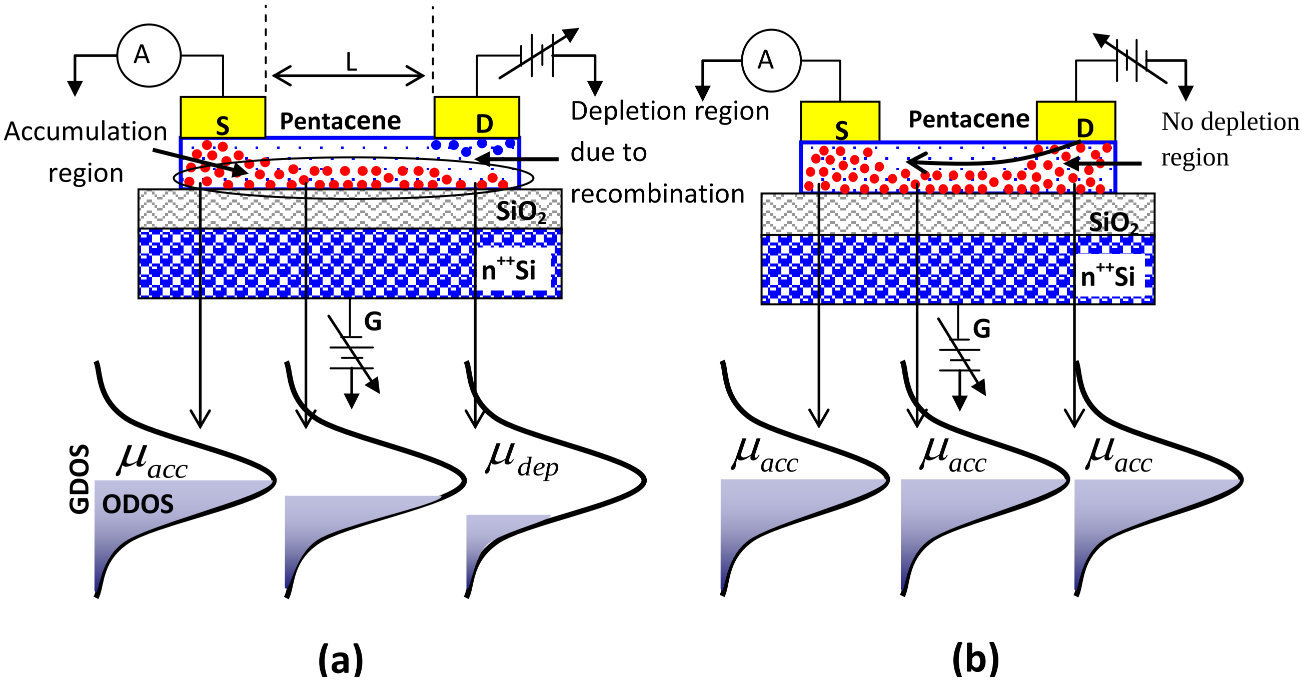

Figure S4. Schematic representation of the working of a p-type OFET in negative VDS regime (left panel) and positive V_{DS} regime (right panel). In negative V_{DS} regime, pinching-off the channel decreases the occupational density of states (ODOS) at the drain contact. In positive V_{DS} regime, as there is no pinch-off, ODOS remain same for the whole channel length.

S6. Variation of Fermi level in negative and positive V_{DS} regime

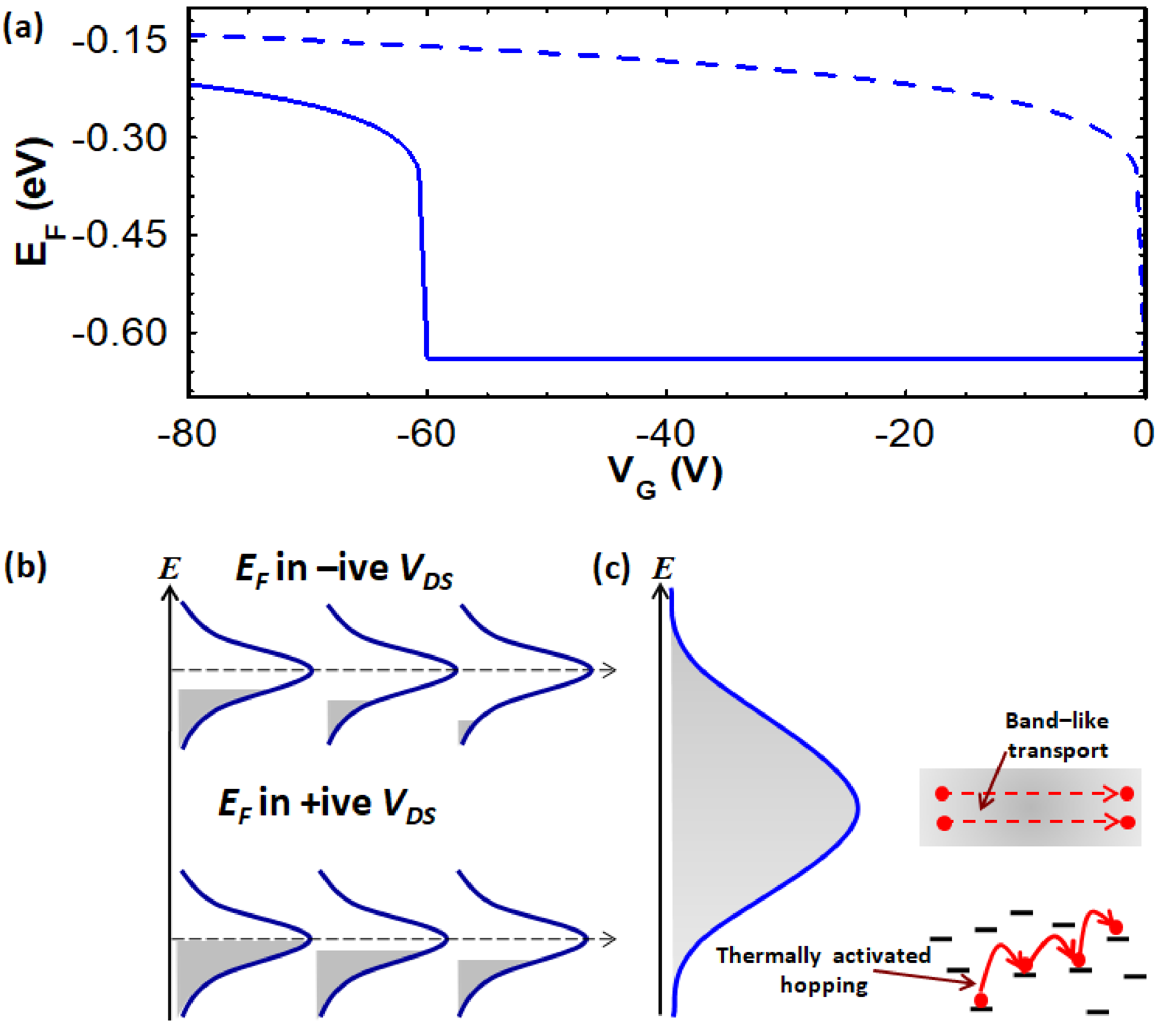

Figure S5: (a) Numerically estimated Fermi level as a function of gate voltage for a fixed negative and positive V_{DS} of 60 V. In negative V_{DS} regime, Fermi level goes down and becomes constant after pinching-off the channel (solid line). However, Fermi level remains high at drain in positive V_{DS} (dashed line). (b) Schematic illustration of position of Fermi level negative and positive V_{DS} regime. (c) Schematic illustration of charge transport governed by two different sections of the Gaussian density of states (GDOS). When the Fermi level lies near the tail of the GDOS, transport is described by thermally activated hopping. As the Fermi level moves towards the central region, band-like transport becomes possible.

References

1. S. Zorba, Y. Shapir, and Y. Gao, Phys. Rev. B **74**, 245410 (2006).

2. H. Yoshida, K. Inaba, and N. Sato, Appl. Phys. Lett. **90**, 181930 (2007).

3. J. Gotzen, D. Kafer, C. Woll and G. Witte, Phys . Rev. B **81**, 085440 (2010).

4. R. Agrawal, P. Kumar, S. Ghosh, and A.K. Mahapatro, Appl. Phys. Lett. **93**, 073311 (2008).

5. A.K. Mahapatro and S. Ghosh, Appl. Phys. Lett. **80**, 4840 (2002).

6. A. Sharma, P. Kumar, B. Singh, S.R. Chaudhuri, and S. Ghosh, Appl. Phys. Lett. **99**, 39 (2011).

7. S. M. Sze and K. K. Ng, Physics of Semiconductor Devices, 3rd ed.; Wiley India, 2010.

8. D. A. Neamen, Semiconductor Physics and Devices, 3rd ed. (Tata McGraw- Hill, New Delhi, 2007).
